# Control of Properties through Hydrogen Bonding Interactions in Conjugated Polymers

**DOI:** 10.1002/advs.202305356

**Published:** 2023-11-09

**Authors:** Qingpei Wan, Barry C. Thompson

**Affiliations:** ^1^ Department of Chemistry and Loker Hydrocarbon Research Institute University of Southern California Los Angeles CA 90089‐1661 USA

**Keywords:** conjugated polymers, hydrogen bonds, organic electronics

## Abstract

Molecular design is crucial for endowing conjugated polymers (CPs) with unique properties and enhanced electronic performance. Introducing Hydrogen‐bonding (H‐bonding) into CPs has been a broadly exploited, yet still emerging strategy capable of tuning a range of properties encompassing solubility, crystallinity, electronic properties, solid‐state morphology, and stability, as well as mechanical properties and self‐healing properties. Different H‐bonding groups can be utilized to tailor CPs properties based on the applications of interest. This review provides an overview of classes of H‐bonding CPs (assorted by the different H‐bond functional groups), the synthetic methods to introduce the corresponding H‐bond functional groups and the impact of H‐bonding in CPs on corresponding electronic and materials properties. Recent advances in addressing the trade‐off between electronic performance and mechanical durability are also highlighted. Furthermore, insights into future directions and prospects for H‐bonded CPs are discussed.

## Graphical abstract

Introducing hydrogen‐bonding (H‐bonding) groups into conjugated polymers (CPs) is a widely employed, yet still emerging strategy effective for tuning a variety of properties. This review addresses recent progress of using H‐bonding in CPs including synthetic methods for introducing the H‐bonding functional groups and the impact on the electronic and materials properties. Prospects for future development are highlighted.

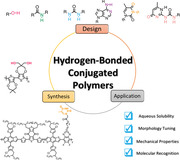

## 1 Introduction

Conjugated polymers (CPs) are a type of polymer with optical, semiconducting/conducting, and/or electrochemical properties that have been developed for use in a number of electronic applications. They are a low‐cost organic materials family that can be solution processed, suitable for roll‐to‐roll (R2R) production, and can be used in lightweight, flexible, and stretchable device applications.^[^ ^1^, ^2^, ^3^, ^4^, ^5^ ^]^ The electrical and physical properties of CPs can be easily modified based on their synthetic tunability. With synthetic customization, physical properties, such as solubility and crystallinity as well as electrical properties including charge transport and light absorption, can be regulated to satisfy the corresponding application of interest. This has led to their successful use in applications such as organic photovoltaics (OPV),^[^ ^6^, ^7^, ^8^, ^9^ ^]^ organic field effect transistors (OFET),^[^ ^10^, ^11^, ^12^ ^]^ organic light emitting diodes (OLED),^[^ ^13^ ^]^ electrochromic devices,^[^ ^14^ ^]^ organic electrochemical transistors (OECT),^[^ ^15^, ^16^, ^17^ ^]^ chemical sensors,^[^ ^18^ ^]^ biological applications,^[^ ^19^, ^20^, ^21^, ^22^, ^23^ ^]^ and photocatalysis.^[^ ^24^, ^25^, ^26^, ^27^ ^]^

Hydrogen bonds (H‐bonds), as a class of noncovalent/secondary bonds, are a special type of dipole–dipole attraction formed by a hydrogen atom lying between two strongly electronegative atoms. A hydrogen atom can be shared between a covalently bonded donor (X) and a free acceptor (Y) with electron lone pairs and H‐bonding is typically denoted as X‐H···Y.^[^ ^28^ ^]^ The most common (X, Y) atoms that can participate in H‐bonds are N, O, and F. The energy of an H‐bond typically can range from 1 to 40 kcal mol^−1^ and is influenced by the geometry, environment, and nature of the participating donor and acceptor atoms.^[^ ^29^ ^]^ H‐bonds have dynamic properties owing to their reversible bonding associations and broadly adjustable binding affinities.^[^ ^30^ ^]^ From simple water molecules to delicate biological macromolecules, H‐bonds, as the most common noncovalent interactions, occur in nature (e.g., DNA, proteins, and carbohydrates).

Molecular design based on H‐bonds has been successfully applied to synthetic materials, such as elastomers, organic framework functional materials, and electroactive polymers, leading to the remarkable development of synthetic H‐bonding materials.^[^ ^31^, ^32^, ^33^, ^34^ ^]^ Elastomers are a type of material that can be dramatically deformed when subjected to an external force and partially or entirely recover if the stress is removed. Elastomers often face repeated stresses, resulting in unexpected degradation, cracking, and even macroscopic fracture. The ability of elastomers to self‐heal is crucial to extend service life time and improve their use safety. Hydrogen bonding plays an important role in the design of self‐healing electroactive elastomers due to the intrinsically dynamic nature and self‐healing elastomers based on multiple hydrogen‐bonding interactions can largely recover to their initial mechanical properties and electrical performance.^[^ ^35^, ^36^, ^37^, ^38^, ^39^ ^]^ H‐bonded organic frameworks (HOFs) are a novel class of porous crystalline materials that self‐assemble from organic or metal–organic building blocks through intermolecular hydrogen‐bonding interactions and HOFs offer several unique characteristics such as mild synthesis conditions, solution processability, self‐healing, and regeneration as H‐bonds are weaker than the coordinate and covalent bonds utilized to produce metal–organic frameworks (MOFs) and covalent organic frameworks (COFs).^[^ ^33^ ^]^ Thanks to the flexible and highly reversible nature of hydrogen bonds, HOFs can be used as a customizable platform for the development of functional materials with significantly increased structural diversity in many applications, such as fluorescent sensing, gas separation and storage, heterogeneous catalysis, and membrane‐based applications.^[^ ^33^ ^]^

Introducing H‐bonding into CPs has been a broadly exploited strategy since H‐bonds can tune a range of properties from solubility and crystallinity, to electronic properties, and morphological stability, as well as mechanical and self‐healing properties.^[^ ^32^, ^40^, ^41^ ^]^ Different H‐bonding group are utilized to tailor the properties that benefit the corresponding application of interest. Polar H‐bonds can promote aqueous solubility of CPs and facilitate polymer‐aqueous phase interaction.^[^ ^42^, ^43^, ^44^, ^45^ ^]^ As a type of directional intermolecular interaction, H‐bonds can significantly affect the conformation as well as optical and physical properties of the polymers involved. For instance, H‐bonds can assist molecules to self‐assemble, offering the material a more ordered and crystalline structure in the solid state. Different levels and motifs of crystallinity will result in significantly different electronic properties as can be advantageous or detrimental depending on the given application. For instance, OFETs benefit from a more aligned and crystalline structure that can enhance the charge transport, whereas OPVs require a mixed amorphous phase.^[^ ^46^, ^47^ ^]^ Stable solid‐state morphology has also been an important parameter for devices to maintain excellent performance under external stimuli, such as temperature fluctuations. H‐bonds embedded in the solid‐state can act as physical cross‐link sites to lock the morphology thus maintaining the initial and optimal morphology.^[^ ^48^, ^49^ ^]^

Due to the reversible nature of H‐bonds, physical cross‐link sites are also capable of acting as energy dissipation centers to absorb external mechanical stress and thus improve the mechanical reliability.^[^ ^50^ ^]^ A central challenge in CPs is to combine excellent electrical performance with robust mechanical reliability. A few representative examples demonstrate the effectiveness of using H‐bonds to address this important trade‐off in organic electronics. Bao et al. utilized amide containing spacers in CPs to realize OFETs with a high mobility of 1.12 cm^2^ V^−1^ s^−1^ at 100% strain along the direction perpendicular to the strain.^[^ ^50^ ^]^ Kim et al. found an amide spacer incorporated CP donor enabling for intrinsically stretchable organic solar cells (IS‐OSC) with a high power conversion efficiency (PCE) of 12.7% and excellent stretchability (PCE retention of >80% of the initial value at 32% strain).^[^ ^51^ ^]^ Likewise, Thompson et al. reported a thymine incorporated fully conjugated CP donor that further improved the PCE of IS‐OSC to 13.7% with a PCE retention of >80% of the initial value at 43% strain, which significantly exceeded the 30% applied stress requirement for wearable electronics.^[^ ^52^ ^]^

**Figure 1:**
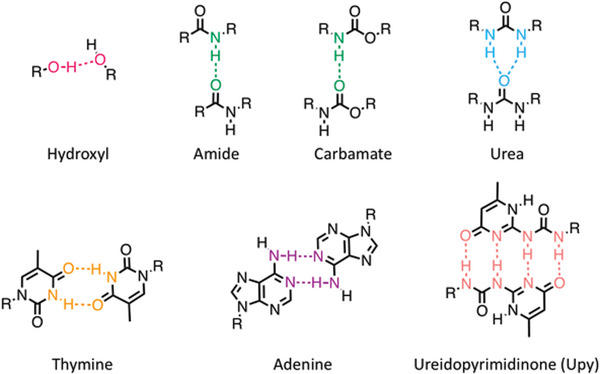
Self‐complementary H‐bond functional groups applied in CPs.

Although a few reviews examining the scope of H‐bonding in CPs have been published, most heavily focus on one specific application and the corresponding device performance.^[^ ^32^, ^40^ ^]^ In this review, recent advances in H‐bonded CPs (assorted by the different H‐bond functional groups, as shown in **Figure** **1**) are broadly reviewed, including the synthetic method to introduce the corresponding H‐bonding functional groups, as well as the impact of H‐bonding on the corresponding materials properties.

Other than self‐complementary hydrogen bonding in Figure 1, specific‐complementary hydrogen bonding between distinct, mutually interacting groups is also an important category. This is analogous to the base‐pairing hydrogen bonding that defines the structure of DNA and RNA. For instance, thymine (Thy) and diaminopyrazine (Dap) is a pair explored in synthetic systems. Thy and Dap display specific‐complementary H‐bonding and are able to form strong multipoint H‐bonds, which have been widely used in supramolecular assembly and molecular recognition.^[^ ^53^, ^54^, ^55^ ^]^ Although specific‐complementary hydrogen bonding has been applied in CPs, only a few examples exist, which will briefly be discussed in the thymine section.

## 2 Common H‐Bonding Functional Groups in CPs

### 2.1 Hydroxyl Group

#### 2.1.1 Overview of the Hydroxyl Group and Representative CPs

**Figure 2:**
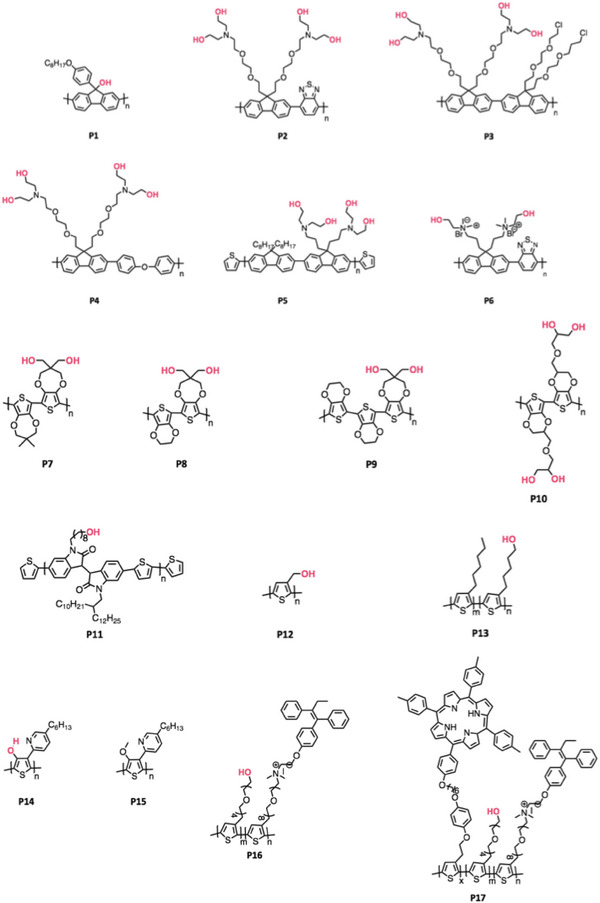
Representative hydroxyl functionalized CPs (P1–P17).

Hydroxyl groups are the simplest functional group that exhibits H‐bonding effects and consists of an oxygen atom which with two lone pairs covalently bonded to a hydrogen atom. The oxygen atom with strong electronegativity can act as the H‐bond donor (X) for the H bond definition X‐H···Y. The oxygen atom of another hydroxyl group can act as the H‐bond acceptor (Y). Alcohols and phenols are representative examples. If other electronegative atoms are present in the system, it is also possible to function as the H bond acceptor for hydroxyl groups.^[^ ^48^ ^]^ Generally, introducing hydroxyl groups into CPs significantly increases the hydrophilicity of the polymer which will benefit applications that require interaction with aqueous media.^[^ ^42^, ^43^, ^44^ ^]^ The most common location of the hydroxyl group in CPs is in the side chain and representative hydroxyl incorporated CPs are shown in **Figure** **2**.

#### 2.1.2 Synthetic Approaches for Introducing Hydroxyl Groups into CPs

Postpolymerization functionalization is the most common strategy used to incorporate hydroxyl groups into CPs. Jen et al. synthesized **P2‐P4** by Suzuki polymerization to form a precursor fluorene polymer with terminal chlorines on the side chains and followed by treatment with diethanolamine to perform the substitution to realize the target polymers.^[^ ^43^ ^]^ Wang et al. reported the fluorene homopolymer **P5** with a similar strategy using a precursor polymer with terminal bromide as the reactive site.^[^ ^42^ ^]^ Kim et al. synthesized the fluorene polymer **P6** with an ethyl hydroxyl trialkyl ammonium salt via the quaternarization of bromoethanol with the precursor polymer containing the tertiary amine.^[^ ^56^ ^]^

**Figure 3:**
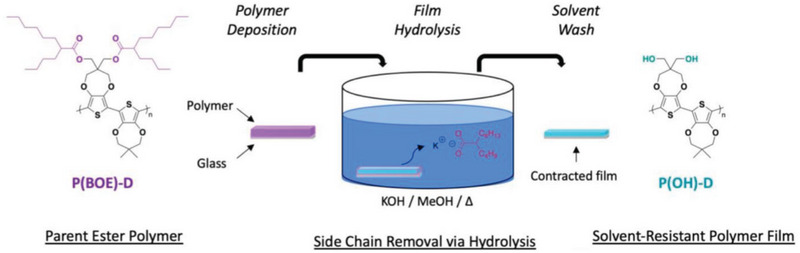
Overview of the conversion of a film of a parent ester polymer to a hydroxyl polymer. Reproduced with permission.^[^ ^44^ ^]^ Copyright 2022, American Chemical Society.

In an alternative postpolymerization strategy, Reynolds et al. synthesized the parent ester polymer of **P7‐P9** by Direct Arylation Polymerization (DArP) under the Fagnou‐derived condition (Pd(OAc)_2_/K_2_CO_3_/PivOH/DMAc).^[^ ^57^ ^]^ The parent ester polymer was cast on a glass substrate and the hydroxyl group was generated via ester hydrolysis using a strongly basic KOH solution to form **P7‐P9**, as shown in **Figure** **3**.^[^ ^44^ ^]^ Rondeau‐Gagné et al. reported the asymmetric hydroxyl group incorporated isoindigo‐based copolymer **P11**.^[^ ^58^ ^]^ The *tert*‐butyldimethylsilyl (TBS) group protected parent polymer of **P11** was synthesized by Stille polymerization and was deprotected under mild acidic condition yielding the hydroxyl group. Similarly, Katz et al. synthesized **P12** by Grignard metathesis (GRIM) polymerization with the TBS protected monomer tert‐butyl(2,5‐dibromothiophen‐3‐6)methoxy)‐dimethylsilane and deprotected to form the hydroxyl group after the polymerization.^[^ ^59^ ^]^ Qiu et al. protected the side chain hydroxyl group by tetrahydropyran (THP) and synthesized parent diblock copolymers (BCPs) via GRIM polymerization followed by deprotection to form the poly(3‐hexylthiophene)‐*b*‐poly[3‐(6‐hydroxy)hexylthiophene] (P3HT‐b‐P3HHT) BCPs (**P13**).^[^ ^60^ ^]^ Kawai et al. synthesized the precursor polymer **P15** via GRIM polymerization and treated with BBr_3_ to carry out the transformation from the methoxy group to the hydroxyl group (**P14**).^[^ ^61^ ^]^

Interestingly, there exist a few CPs synthesized by directly polymerizing monomers bearing hydroxyl groups. Huang et al. reported the synthesis of **P1** and similar fluorene polymers without hydroxyl groups by Yamamoto polymerization and were able to achieve number‐average molecular weight (*M*_n_) of 24.7 kg mol^−1^.^[^ ^62^ ^]^ Wang et al. conducted oxidative copolymerization with hydroxyl terminated thiophene in the presence of FeCl_3_ to give **P16**.^[^ ^63^ ^]^ Luo et al. grafted glycidol onto the poly(3,4‐ethylenedioxythiophene (EDOT) and electrochemically polymerized on glassy carbon electrodes (GCE). ^[^ ^64^ ^]^

Ultimately, postpolymerization functionalization has the advantage of excluding the effect of the hydroxyl group during the polymerization, so different types of polymerizations, such as GRIM, Suzuki, Stille, and DArP have been applied to hydroxyl incorporated CPs precursor polymer synthesis. Additionally, the quality of the polymer, such as molecular weight and defects should be nearly identical to the precursor polymer. However, the reaction of converting to the hydroxyl group after polymerization might be a tedious step, especially when complete conversion is needed. Considering that most polymer NMR signals are broad and offer limited information, in most of the cases, the presence of the hydroxyl group after postpolymerization reaction is qualitatively (not quantitatively) confirmed.

#### 2.1.3 Materials Properties of Hydroxyl Group Functionalized CPs

After introducing the hydroxyl group, CPs typically exhibit poor solubility in nonpolar aromatic solvents, such as toluene compared to CPs without the hydroxyl group. A more polar solvent such as THF or DMF often provides good solubility for the hydroxyl group functionalized CPs, due to the formation of hydrogen bonds between the polymer and solvent. Huang et al. utilized this property and prepared an H‐bonded CP gel (**P1**/toluene gels via supramolecular self‐assembly behavior) by a heating‐cooling process where a **P1** toluene solution with a concentration of 25 mg mL^−1^ was heated to 80 °C for 10 min and then cooled to room temperature by standing for 30 h.^[^ ^62^ ^]^ Huang et al. conducted a solvent effect study on **P1** gel formation and found the solvent immobile organogel was able to form in nonpolar or low polarity solvents, such as dichloromethane (DCM), chloroform (CHCl_3_), 1,2‐dichloroethane (DCE), toluene, bromobenzene, chlorobenzene, and 1,2‐dichlorobenzene, but was not able to form in polar aprotic solvents, such as DMF, 1,4‐dioxane, and THF.^[^ ^62^ ^]^ Additionally, Huang et al. showed that the emission color of **P1** supramolecular thin films can be dynamically tuned from blue to yellow via selecting different types of solvent and *M*_n_ because these two factors significantly affect the aggregation of **P1**.^[^ ^62^ ^]^

Hydroxyl functionalized CPs combine the advantages of conjugated polyelectrolytes and traditional neutral surfactants,^[^ ^43^ ^]^ which allow them to be processed from environmentally‐friendly alcohol solutions. The polar groups on their side chains can also facilitate electron injection from high work‐function metal cathodes because hydroxyl groups can interact with high work‐function metals to form a positive interfacial dipole between the cathode and the electron transporting layer (ETL), which results in a reduced injection barrier at the interface.^[^ ^43^ ^]^ Jen et al. developed **P2‐P4** with different electron donating/withdrawing monomers and applied them as electron injecting layers in Polymer light‐emitting diodes (PLEDs).^[^ ^43^ ^]^ Owing to the advantage of the polar hydroxyl group and the interaction with the metal electrode, these polymers modified the work function (WF) of the metal electrode, aligning the energy levels of the electrode, and active layer in organic photovoltaics (OPV) which ensures energy level alignment for effective charge extraction. Additionally, the issue of the difference between the hydrophilic metal surface and the hydrophobic OPV active layer can be addressed because the polymer interlayer can reduce interfacial tension. Wang et al. combined the active layers including high‐performing acceptor and donor polymers (all polymer solar cells) with **P5** as an interlayer and found the power conversion efficiency (PCE) was increased from 2.7% (without **P5**) to 5.3% (with **P5**), which is comparable with the conventional devices with LiF/Al.^[^ ^42^ ^]^ More interestingly, Kim et al. found that **P6**, as the interfacial layer, led to an even greater PCE improvement than the nonhydroxyl star interlayer polymer PFN‐BT because the OPV device with **P6** had a lower series resistance. Specifically, OPV devices with PTB7‐Th as the donor and PC_71_BM as the acceptor and **P6** as the interfacial layer exhibited an average PCE of 10.5%, while the same device with PFN‐BT exhibited an average PCE of 9.6%.^[^ ^56^ ^]^

In addition to enabling interfacial layers, hydroxyl groups can also can play a critical role in CP doping. Ponder Jr. et al. reported that the electrical conductivity of chemically doped CP films was significantly increased after postprocessing side chain removal of the parent ester polymers of **P7‐P9** and demonstrated the increase in electrical conductivity is mainly due to an increase in charge carrier density and reduction in carrier localization that occurs after side chain removal.^[^ ^44^ ^]^ The polarity of the hydroxyl groups on **P7‐P9** also offers aqueous electrochemical compatibility. Impressively, **P9** exhibits an exceptional electrical conductivity (≈700 S cm^−1^), which is better than all previously reported glycol‐based CPs. Additionally, Reynolds et al. showed that short hydroxyl substituents (**P7**) can afford facile doping and high volumetric capacitance (C*) in saline‐based electrolytes and long polar side chains are not required.^[^ ^45^ ^]^ The hydroxyl groups on the side chain can act as both hydrogen bond donors and acceptors. The hydrogen bonds formed in aqueous media can benefit the polymer–electrolyte interactions and facilitate the uptake of hydrated ions, which might induce special polymer–electrolyte interactions in aqueous media that are not observed for the other glyme side chains.^[^ ^45^ ^]^ Therefore, **P7** has the highest C* (106 ± 7 F cm^−3^) across the entire voltage range compared to other glyme side chains polymers.

Luo et al. designed and synthesized a novel conducting polymer **P10** by electrochemical polymerization.^[^ ^64^ ^]^ The excellent antifouling properties of the surface of **P10** were demonstrated by cell attachment studies with both human cervical carcinoma (HeLa) cells and Michigan Cancer Foundation‐7 (MCF‐7) cells. Nearly full coverages of HeLa and MCF‐7 cells were observed on PEDOT surfaces, whereas a very limited number of cells attached to the PEDOT‐HPG (**P10**) surfaces, which showed the PEDOT‐HPG (**P10**) surface can effectively resist the nonspecific cell attachment. Luo et al. proposed that the good antifouling capability mainly arises from the prominent hydrophilicity due to the presence of glycol groups on the polymer, which helps to form a hydration layer between proteins and the electrode surface thus creating a barrier that inhibits the adsorption of proteins and other contaminants.

Rondeau‐Gagné et. al. reported the isoindigo‐based polymer **P11** with improved processability in alcohol‐based solvents. **P11** demonstrated the highest average mobility (2.49 × 10^−4^ cm^2^ V^−1^ s^−1^) when processed in 20% v/v o‐anisole/n‐BuOH in thin film organic field‐effect transistors thanks to the hydroxyl moieties.^[^ ^58^ ^]^ Additionally, **P11** thin film coupling with an fluorescein isothiocyanate (FTIC) probe using dibutyltindilaurate demonstrated that the terminal hydroxyl groups are capable of solid‐state postfunctionalization toward the development of multifunctional organic electronics.^[^ ^58^ ^]^

Katz et al. studied the sensing properties of **P12** as a bioreceptor in organic electrochemical transistors (OECT) since hydroxyl groups target hydrogen bonds between the polymer films and biomolecules, which can aid the immobilization of the biomolecules and create larger sensing signals.^[^ ^59^ ^]^ Although the sensitivity of **P12** is relatively small, it exhibits better specificity since the smaller Vth change for both the antihuman Immunoglobulin G (IgG) and myelin basic protein (MBP) pair and bovine serum albumin (BSA) and IgG pair has been observed and the signal change of pure **P12** only comes from the specific binding between antibody and antigen.

In addition to homopolymers and alternating copolymers, hydroxyl groups have also been applied to block copolymers. Qiu et al. synthesized **P13** BCPs with different block ratios which can be cross‐linked since hydroxyl groups are crosslinked during thermal annealing by releasing water.^[^ ^60^ ^]^ The obtained BCPs formed microphase separated structures due to the different polarities of the two blocks. After thermal annealing at 200 °C, the cross‐linking of the hydroxyl block disturbed the microphase separated structure and the roughness of films increased and the degree of crystallization greatly improved, which is caused by the rearrangement of the non‐crosslinked parts. It was also demonstrated that cross‐linking during thermal annealing at 200 °C not only improved the degree of crystallization but also the ductility of films.^[^ ^60^ ^]^ Qiu et al also investigated the crystallization, microphase separation and photophysical properties of **P13** BCPs in mixed solvents.^[^ ^65^ ^]^ After adding 20% methanol into pyridine solution, nanofibers were observed. When the volume ratio of methanol/pyridine was 40: 60, the nanofibers disappeared and ordered spherical micelles started to be seen. Since methanol is a poorer solvent for the hydrocarbon side chain block than the hydroxyl side chain, the block with hydroxyl groups was more swollen and became larger in volume than hydrocarbon block while adding methanol. Eventually, the BCPs transformed into spherical micelles with the hydrocarbon side chain block as the core surrounded by the hydroxyl side chain block corona to minimize the interfacial energy. Further increasing the ratio to 70: 30, the spherical micelles aggregated to a much larger size.

**Figure 4:**
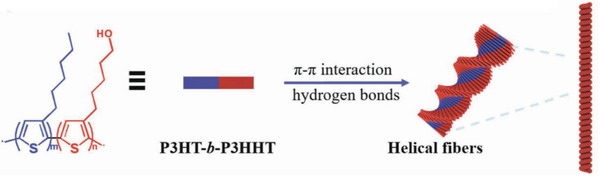
Schematic illustration of the formation of helical nanofibers in **P13**. The blue and red colors represent the P3HT block and P3HHT block, respectively. Reproduced with permission.^[^ ^66^ ^]^ Copyright 2018, Royal Society of Chemistry.

Peng et al prepared 1D helical nanofibers through the self‐assembly of **P13** in an aged pyridine solution and proposed that such helical nanofibers were formed by the π–π interaction between rigid polythiophene backbones plus the hydrogen‐bonding interactions between the polar hydroxyl groups of the side chains, as shown in **Figure** **4**.^[^ ^66^ ^]^ More interestingly, the Young's modulus of such helical fibers is about 5.16 GPa, which is about two times higher than the P3HT films characterized by the peak force quantitative nanomechanical (PF‐QNM) method and the field effect mobility of these helical fibers is as high as 0.034 cm^2^ V^−1^ s^−1^.

Temperature‐dependent infrared (FTIR) spectroscopy measurements were conducted to prove hydrogen bonding in the solid state polymer film of **P13**.^[^ ^66^ ^]^ The *─*OH stretching vibration of the initial film appeared at 3335 cm^−1^ and gradually blueshifted to the vibration peak at 3495 cm^−1^ as the temperature increased, which was mainly due to the splitting of the hydrogen bonds and hydroxyl groups becoming free *─*OH groups.^[^ ^67^ ^]^

Takagi et al. supported the intramolecular hydrogen bonding between pyridine and the hydroxyl groups via DFT calculations on model compounds and found a significant redshift of the absorption maxima from 476 nm (**P15**) to 662 nm (**P14**), which is mainly caused by formation of intramolecular hydrogen bonding.^[^ ^61^ ^]^ Wang et al. designed and synthesized **P16** and **P17** polythiophene‐tamoxifen conjugates for intracellular molecule‐targeted binding and inactivation of protein for growth inhibition of MCF‐7 cancer cells by incorporating the small molecule drug into the side chain of the conjugated polymer.^[^ ^63^ ^]^ The hydroxyl side chain thiophene moiety not only acted as the key reactive site to be converted into other important functional group (i.e., Tamoxifen) but also can improve the hydrophilicity when it is on the polymer chain.

### 2.2 Amide and Carbamate Groups

#### 2.2.1 Overview of Amide and Carbamate Groups and Representative CPs

Secondary amides are the most commonly used amide groups to introduce H bonding into polymers because the hydrogen atom in the *─*NH group is positive enough to form a H bonding with a lone pair on the oxygen atom of another amide group (the N atom covalently bonded with the H serves as the H bonding donor X and the O atom from the carbonyl group of a different secondary amide will serve as the H bonding acceptor Y in the scheme X‐H···Y). Generally, introducing an amide or carbamate group into CPs can effectively tune features such as crystallinity, molecular packing, and mechanical properties of the polymer. Most commonly amide and carbamate groups are introduced into the side chain of CPs and representative amide and carbamate incorporated CPs are shown in **Figure** **5**.

Figure 5Representative amide and carbamate functionalized CPs (P18–P45).

**Figure:**
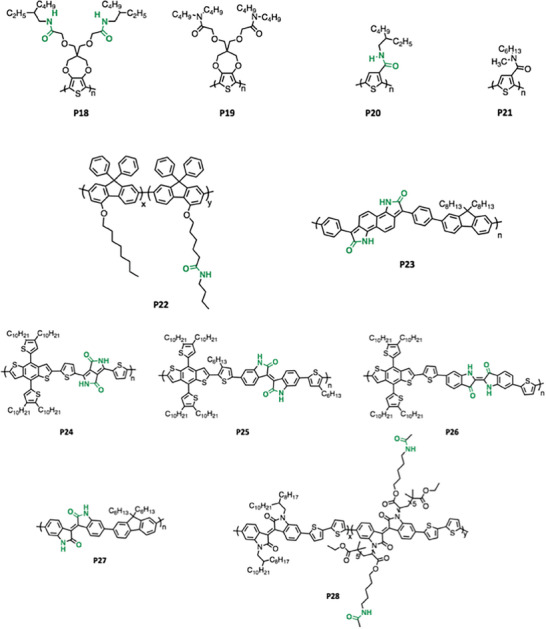

**Figure:**
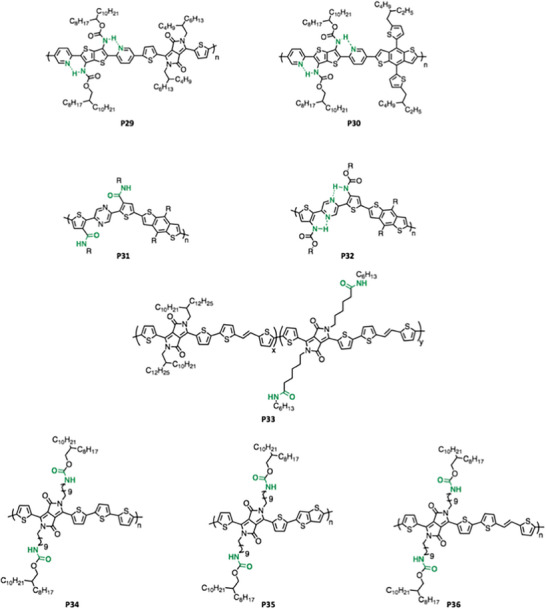

**Figure:**
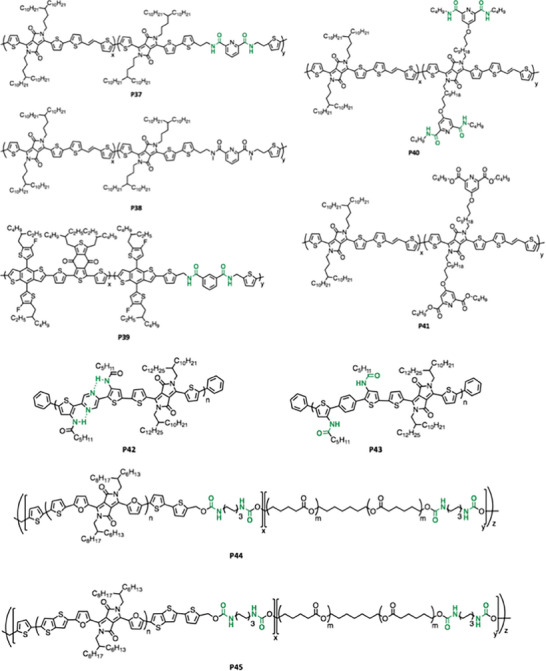

#### 2.2.2 Synthetic Approaches for Introducing Amide and Carbamate Groups into CPs

Generally, the synthesis of amide and carbamate functionalized monomers followed by polymerization is the most common strategy for incorporation into CPs and most often the amide or carbamate group is found on an aryl‐halide monomer. Specifically, this corresponds to the two polymerization strategies of terpolymerization and perfectly alternative polymerization. In the terpolymeization approach, typically a benchmark polymer serves as the parent polymer and an amide or carbamate functionalized monomer is used as the third monomer. The content of group incorporated is directly tuned by the ratio of the amide or carbamate containing monomer that is added into the polymerization and representative polymers include **P28**, **P33**, **P37**‐**P38**, and **P39**‐**P41**.^[^ ^50^, ^51^, ^68^, ^69^, ^70^ ^]^ Most of these polymers are achieved by traditional cross‐coupling polymerization such as Suzuki or Stille polymerization under conditions very similar to the parent polymer since the ratio of the incorporated amide or carbamate monomer is generally relatively low (from 5 to 30% mol). For instance, Huang et al. synthesized **P22** by Yamamoto polymerization.^[^ ^71^ ^]^ A very clear trend from the synthesis of these polymers is that nearly all of the amide or carbamate polymers with higher loading (e.g., ≥ 20% mol) have a lower *M*_n_ than the analogous polymers with lower loading (e.g., ≈5%mol). As the ratio of the amide or carbamate monomer is increased, the solubility very likely decreases. Typically, an optimal and balanced ratio (between 5 and 30% mol) is used considering the application of the polymers.

**Figure 6:**
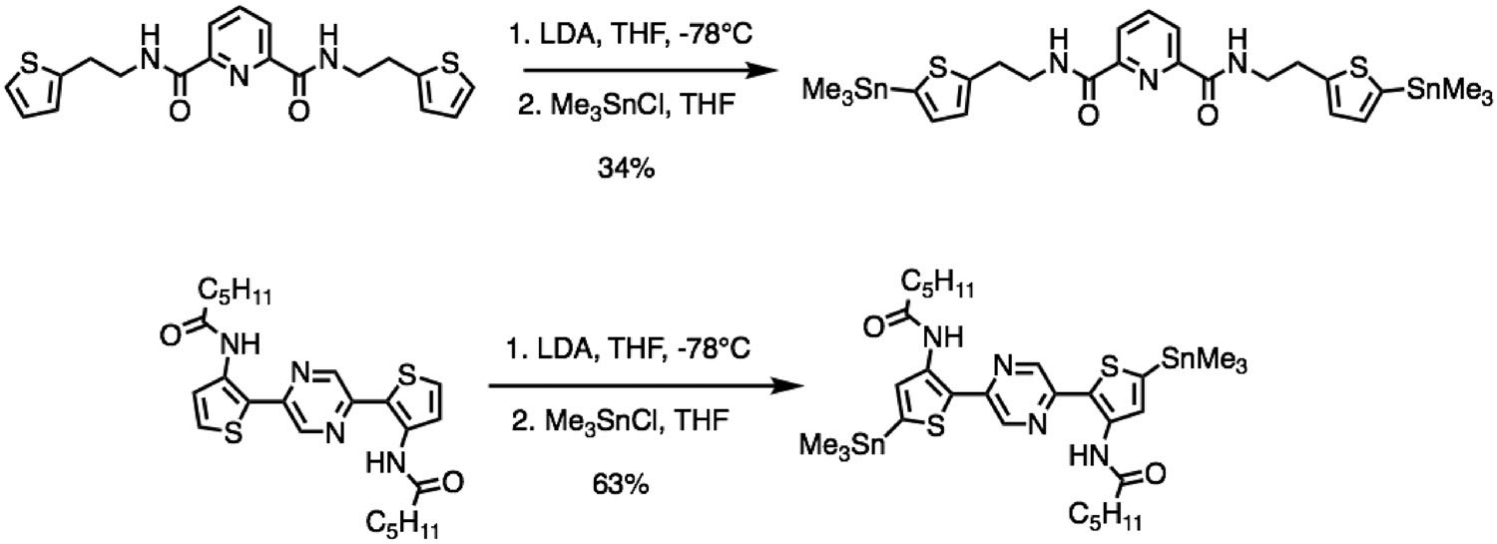
Stannylation of the thiophene with secondary amide.^[^ ^50^, ^75^ ^]^

A perfectly alternating polymerization strategy includes the amide or carbamate functionalized monomer as the only comonomer such as **P29**‐**P32**, **P34**‐**P36**, and **P42‐P43**.^[^ ^72^, ^73^, ^74^, ^75^ ^]^ It is worth noting that the amide and carbamate functionalized perfectly alternative polymers **P29**‐**P32** and **P34**‐**P36** all have a reasonable molecular weight (with *M*_n_ higher than 50 kg mol^−1^). **P42** and **P43** have lower molecular weight (10–15 kg mol^−1^) and this might be due to the end capping effect which introduces monobromo or monostannyl compound to stop the chain growing. Significantly, Bao et al. and Rondeau‐Gagné et al. were able to conduct the stannylation of thiophene via lithium diisopropylamide (LDA) in the presence of the N*─*H bond in the secondary amide, as shown in **Figure** **6**.^[^ ^50^, ^75^ ^]^

Postpolymerization functionalization is another method used to introduce amide and carbamate groups into CPs. Huang et al. synthesized *tert*‐butoxycarbonyl (*t*‐Boc) substituted indigo, isoindigo, and diketopyrrolopyrrole (DPP) acceptor units and conducted the thermal treatment of **P24‐P26** films at 200 °C for 10 min to deprotect the *t*‐Boc side groups and form the amide groups.^[^ ^76^ ^]^ Thermogravimetric (TGA) analysis indicated a two‐step thermal decomposition of these copolymers and the first weight loss occurred at ≈190 °C which arose from the elimination of the *t*‐Boc groups. FT‐IR spectroscopy indicated the original stretching vibration band of C═O from *t*‐Boc at ≈1700 cm^−1^ disappeared and the new characteristic band of C═O of the lactam moiety shifted to slightly smaller wavenumbers, which supports the nearly complete deprotection of the *t*‐Boc groups. Additionally, a new band appeared at ≈3450 cm^−1^ after thermal treatment, which corresponds to the N*─*H···O═C hydrogen bonding resulting from the lactam structures. Zhu et al. utilized a similar postpolymerization method to prepare the **P23** and **P27**.^[^ ^77^, ^78^ ^]^

Oxidative polymerization and DArP have also been utilized to polymerize amide functionalized monomers. Mei et al. conducted FeCl_3_ mediated oxidative polymerization with a ProDOT monomer to obtain polymers **P18** and **P19** although the molecular weight of the resulting polymers is slightly lower than 10 kg mol^−1^.^[^ ^79^ ^]^ Thompson et al. utilized DArP to synthesize **P21** with an *M*_n_ of up to 15.4 kg mol^−1^ and yields of up to 90% by polymerizing the corresponding monomer 5‐bromo‐*N*‐hexyl‐*N*‐methylthiophene‐3‐carboxamide.^[^ ^80^ ^]^ Interestingly, the optimal DArP condition for **P21** cannot be directly applied to secondary amide polymer **P20** and ^1^H NMR studies show impurities in the aliphatic region which can be the result of *N*‐arylation of the secondary amide. Thompson et al. then employed a modified condition using Pd(OAc)_2_ with P(t‐Bu)_2_Me‐HBF_4_ as a ligand and K_2_CO_3_ a base to synthesize **P20** successfully with a satisfactory *M*_n_ (11.6 kDa). Importantly, the ^1^H NMR exhibited no apparent impurity in the aliphatic region, and the N*─*H resonance (δ 5.82 ppm) remains after polymerization. However, H‐bonding between secondary amides likely resulted in a fraction of insoluble polymer that caused a lower yield for the polymerization compared with **P21**.

As an alternative approach, the reaction between a hydroxyl end group and an isocyanate group can form secondary amides in the polymer backbone. For instance, Lipomi et al. synthesized **P44** and **P45** via the polyaddition between a diketopyrrolopyrrole (DPP) block, and the poly(ε‐caprolactone) (PCL) block using DPP diol blocks and hexamethylene diisocyanate.^[^ ^81^ ^]^

#### 2.2.3 Materials Properties of Amide and Carbamate Functionalized CPs

The materials properties of amide and carbamate functionalized CPs can be classified into two categories. The first category is related to engendering aqueous solubility/compatibility since most CPs have a hydrophobic nature but as amide and carbamate groups are polar functional groups, hydrophilic character is introduced. For instance, Mei et al. found that the presence of amide groups in polymer side chains can facilitate redox switching in aqueous electrolytes while preserving a high electrochromic contrast.^[^ ^79^ ^]^ Additionally, the presence of the amide group was found to reduce the oxidation onset from 0.3 to 0.15 V and the absorbance spectra of **P18** exhibited a red‐shifted *λ*_max_ value and absorbance onset compared to the polymer without the amide group, most likely stemming from H‐bonding induced ordering.^[^ ^79^ ^]^ Similar impacts on solubility have also been observed by Thompson et al with demonstration that **P20** and **P21** can be processed using green polar solvents, such as ethanol and 1‐butanol.^[^ ^80^ ^]^ Generally, an extremely high loading or nearly a full loading of amide groups in the side chains is required to realize aqueous solubility. Although both hydroxyl and amide groups were reported for use as functional groups intended to increase aqueous solubility/compatibility, hydroxyl groups are significantly more commonly used than amides. The hydrogen bond generated from hydroxyl groups is typically stronger than the hydrogen bond formed in the amide and carbamate group since the oxygen atom is more electronegative than the nitrogen atom.^[^ ^82^, ^83^ ^]^ Therefore, introducing the hydroxyl group will likely have a more significant impact on the aggregation, crystallinity, and solid state behavior of the polymer than the amide and carbamate group.

The second category is related to using H‐bonding in secondary amides to tune aspects, such as crystallinity and mechanical properties. Huang et al. found that physical cross‐links via interchain H‐bonds were able to facilitate chain entanglement and aggregation in solution via dynamic light scattering (DLS) and rheological measurements. Additionally, increasingly pronounced diffraction peaks were observed in wide‐angle X‐ray scattering (WAXS) as the ratio of the amide functionalized monomer increased indicating that H‐bonds are favorable for promoting long‐range‐order in **P22**.^[^ ^71^ ^]^ Huang et al. also demonstrated that polymers with a higher ratio of secondary amide have better mechanical properties and the enhanced toughness mainly results from the interchain network assisted by H‐bonding interactions and the resulting energy‐dissipation centers derived from the rigid crystalline nanodomain.^[^ ^84^ ^]^

**Figure 7:**
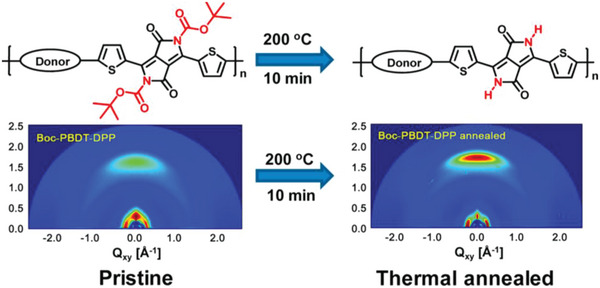
GIWAXS of **P24** before and after annealing at 200 °C for 10 min. Reproduced with permission.^[^ ^76^ ^]^ Copyright 2015, American Chemical Society.

Huang et al. also reported significantly improved field effect hole mobility of **P24‐P26** copolymers after forming the amide group through deprotection of *t*‐Boc side groups.^[^ ^76^ ^]^ The increase in mobility upon annealing is correlated with an increase in the intensity of the sharp reflections (GIWAXS) for the thermally annealed films, which indicates substantially improved intermolecular packing, as shown in **Figure** **7**. H‐bonding from the amide group partially contributes to this improved molecular ordering and improved packing generally induces higher charge carrier mobility. Huang et al. also investigated the photovoltaic performance of bulk heterojunction solar cells by blending the polymers **P24‐P26** with [6,6]‐ phenyl C_71_ butyric acid methyl ester (PCBM) but did not find a significant increase in the power conversion efficiency (PCE) after annealing, which might be due to significant phase separation and coarsening of the film morphology.^[^ ^76^ ^]^ Similarly, Zhu et al. observed a strong bathochromic shift in the UV–vis spectra and narrower bandgap in the H‐bonded polymer **P23** and the electron mobility of **P23** was 0.01 cm^2^ V^−1^ s^−1^ which was about 40 times higher than the precursor with a mobility of 2.4 × 10^−4^ cm^2^ V^−1^ s^−1^.^[^ ^77^ ^]^ Zhu et al. also applied this strategy to an isoindigo polymer.^[^ ^78^ ^]^

The DPP unit is one of the key building blocks for high performance OFETs since the first thiophene‐flanked DPP‐based polymer semiconductor was reported and showed hole mobility (µ_h_) of 0.1 cm^2^ V^−1^ s^−1^ and electron mobility (µ_e_) up to 0.09 cm^2^ V^−1^ s^−1^, respectively.^[^ ^85^ ^]^ Introducing an H‐bonding unit such as secondary amide into the side chain of DPP unit is a prevailing strategy. Rondeau‐Gagné et al. found that incorporation of a small amount (5 mol%) of DPP monomer with amide side chains (**P33)** enabled a maximum hole mobility of 2.46 cm^2^ V^−1^ s^−1^ in OFET devices.^[^ ^69^ ^]^ Interestingly, Rondeau‐Gagné et al. also found that side‐chain engineering with amide moieties reduced the crystallinity of the DPP polymers in the thin film state, which is in contrast to other polymers, but significantly influenced the mechanical properties of the DPP‐polymers by improving their stretchability and lowering the elastic modulus. **P33** with 10% H‐bonding side chains can be stretched up to 75% elongation without any nanoscale cracks on a PDMS substrate and damaged films could be recovered after chlorobenzene solvent vapor annealing and thermal annealing.^[^ ^86^ ^]^ Oh et al. reported the well‐defined alternating donor–acceptor polymers **P34‐P36** by synthesizing the branched carbamate‐based DPP monomer which not only provided structural regularity with moderate H‐bonding but also guaranteed sufficient solubility.^[^ ^74^ ^]^ Thin films of **P36** demonstrated the highest mechanical stability, maintaining their electrical and molecular packing characteristics under strains of up to 100% and showing a healing property.

Chen et al. incorporated poly (acrylate amide) (PAAm) side chains along with octyldecane (OD) into isoindigo‐bithiophene conjugated copolymers to construct the intrinsically stretchable polymer **P28**.^[^ ^68^ ^]^ The soft and bulky PAAm side chains improved the morphology of the thin film surface under strain and the stretchability and mobility were improved by combining hydrogen bonding with the soft acrylate unit. The experimental results demonstrated that with 5–10% PAAm5 (5 repeat unit of poly (acrylate amide)) improved crystallinity and stretchability were observed but higher numbers of repeat units of PAAm led to poor crystallinity and lower charge carrier mobility due to the bulkiness of the side chains, which disrupted the molecular stacking.

In addition to intermolecular H‐bonding, intramolecular H bonding within the polymer backbone has also been studied using amide groups. Rondeau‐Gagné et al. synthesized **P42** and **P43** by incorporating pyrazine or benzene moieties flanked by thiophenes with pendant amide side chains and the orientation and type of H‐bonds were carefully controlled by using either pyrazine (**P42**) or benzene (**P43**) in the polymer backbone.^[^ ^75^ ^]^ Interestingly, **P42** has a maximum mobility of 0.162 cm^2^ V^−1^ s^−1^, which is three orders of magnitude greater than **P43** and the **P42**‐based OFET devices also had a good *I*_on_/*I*_off_ current ratio (10^5^) and low threshold voltage, which is likely due to the more planar polymer chains and better solid‐state morphology induced by the intramolecular H‐bonds. Surprisingly, despite being more crystalline and more rigid due to the more planar polymer chains, **P42** was found to be a softer material (tensile modulus of 361 MPa) than **P43** (tensile modulus of 501 MPa), which is explained by the presence of intermolecular H‐bonds in **P43** which also act as cross‐linking sites resulting in stiffer materials.

Similarly, Zhang et al. synthesized the pyridine‐thieno[3,2‐b]thiophene‐pyridine building block and the weak intramolecular noncovalent interactions enabled a rigid co‐planar structure with extended π‐conjugation, and a tight lamellar arrangement in the solid state.^[^ ^72^ ^]^ **P29** had a p‐type field‐effect mobility of 0.17 cm^2^ V^−1^ s^−1^ and **P30** based polymer solar cells exhibited a notable power conversion efficiency of 10.8%. Zhang et al. also synthesized the thiophene‐pyrazine‐thiophene building blocks with carbamate substituents where intramolecular hydrogen bonds were able to form within the polymer backbone.^[^ ^73^ ^]^ Interestingly, the PCE of the devices based on **P32** with intramolecular hydrogen bonds was 5–8%, while the PCE of the devices based on **P31** with intermolecular hydrogen bonds was 0.1%, which is attributed to the higher bimolecular recombination, geminate recombination, and reduced face‐on orientation of the blend.

Surprisingly, polymers with similar backbones with intramolecular hydrogen bonding exhibit significantly better performance in both OFET and OPV devices. In OFETs, intramolecular hydrogen bonding functionality in **P42** shows three orders of magnitude greater hole mobility than the intermolecular hydrogen bonding in **P43**. In OPVs, intramolecular hydrogen bonding in **P32** enables a PCE of 5–8%, while the intermolecular hydrogen bonding in **P31** leads to a PCE of only 0.1%. Intramolecular hydrogen bonding likely generates more planar polymer chains and improved solid‐state morphology, which can lead to improved face‐on orientation.

Amide‐based derivatives such as 2,6‐pyridine dicarboxamide (PDCA) have also been introduced into CPs to construct intrinsically stretchable and healable semiconducting layers.^[^ ^50^ ^]^ Bao et al. introduced the PDCA building block to synthesize **P37** (10% mol PDCA) and **P38** (10% mol PDCA) with methylation of amide group as a the reference polymer.^[^ ^50^ ^]^ The reason why PDCA was chosen to introduce H‐bonding within the flexible polymer backbone is because it contains two amide groups with moderate hydrogen‐bonding strength, allowing the formation of a polymer network without significantly increasing the material's tensile modulus. Interestingly, although intermolecular hydrogen bonding was supposed to effectively cross‐link the polymers, which is anticipated to increase the elastic modulus of the polymer film, it appears that reducing the rigidity of the conjugated polymer backbone had a greater effect on the elastic modulus of the polymer semiconductor film and this lead to both **P37** and **P38** having a lower modulus than the perfectly alternating parent DPP polymer.

**Figure 8:**
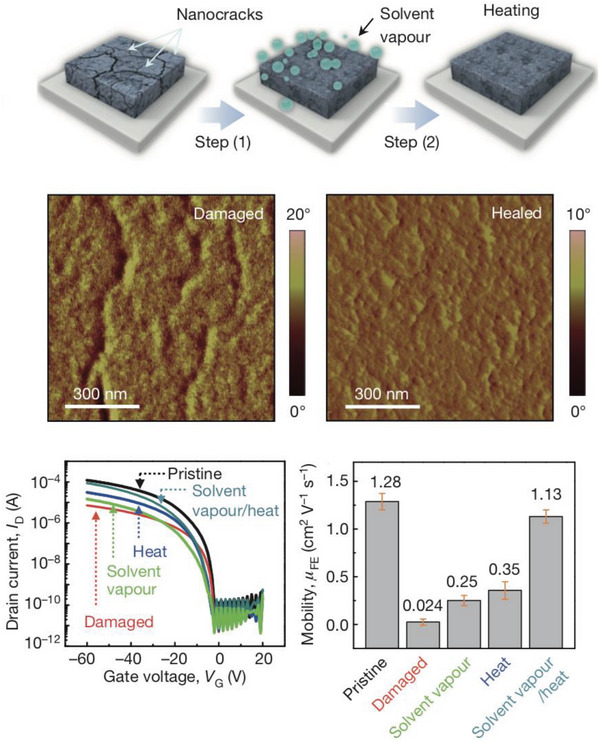
Illustration of the treatments used for healing conjugated polymer films after stretching (top). AFM phase images for damaged and healed films of P37 (middle). Transfer curves and field‐effect mobility of damaged and healed films of **P37** (bottom). Reproduced with permission.^[^ ^50^ ^]^ Copyright 2016, Springer Nature.

Bao et al. found that when applying strains up to 100%, the average field‐effect mobility of **P37** decreased from 1.32 to 0.11 cm^2^ V^−1^ s^−1^ along the direction of applied strain and after releasing the applied strain, the mobility was observed to recover to 1.00 cm^2^ V^−1^ s^−1^.^[^ ^50^ ^]^ When strain was applied perpendicularly, the mobility of **P37** is maintained at >1 cm^2^ V^−1^ s^−1^ even up to 100% strain. As a control, **P38** exhibited decreased stretchability, with a crack onset strain of ≈25%. The healing ability of **P37** was demonstrated via combined thermal and solvent annealing, which promoted the most efficient healing of the polymer films and a complete disappearance of the nanocracks within the damaged films, as well as an almost complete recovery of the average field‐effect mobility to 1.13 cm^2^ V^−1^ s^−1^, as shown in **Figure** **8**.^[^ ^50^ ^]^ Fully stretchable OTFTs based on **P37** were fabricated and the majority of the devices had field‐effect mobilities in the 10^−1^ cm^2^ V^−1^ s^−1^ range with > 10^5^ on/off current ratio.

In order to investigate the electrical performance of the transistors under various mechanical strain conditions to verify their stretchability, the fabricated fully stretchable devices were mounted on human limbs to undergo a series of common movements such as arm folding, hand twisting, and elbow stretching to test the device tolerance. Under all of these conditions, **P37** based fully stretchable OTFTs maintained an average mobility of >0.1 cm^2^ V^−1^ s^−1^.

Bao et al. also inserted the PDCA moiety into the side chain of a DPP‐based CP to synthesize **P40**. By comparing with the reference **P41**, it was determined that PDCA in the side chains produced almost quantitative formation of intermolecular H‐bonding even at low PDCA content (10 mol%).^[^ ^70^ ^]^ Attenuated total‐reflectance Fourier transformation infrared spectroscopy (ATR‐FTIR) was conducted to analyze differences in intermolecular H‐bonding between the PDCA unit in the side chain and the backbone of the polymer since broad IR peaks in the amide region are typically attributed to bound protons, whereas sharp peaks at higher wavenumbers are typically attributed to free NH groups.^[^ ^70^ ^]^

When PDCA units are located in the side chains (**P40**), almost all of the N*─*H signals are in the H‐bonding state (3327 cm^−1^) even at only 10 mol%.^[^ ^70^ ^]^ In comparison, polymers with PDCA units within the backbone only achieve a high degree of bonding at 60 mol% and films made of backbone incorporated PDCA (**P37**) showed a clearer evolution in the intensity and position of the C═O stretching with a higher amount of PDCA in the structure.^[^ ^70^ ^]^ It is also worth noting that pyridine moieties in the PDCA unit may also contribute to improved mechanical properties by participating in intra‐ and intermolecular hydrogen bonding.

Kim et al. synthesized **P39** by introducing the amide incorporated *N* ^1^, *N* ^3^‐bis((5‐bro‐ mothiophen‐2‐yl)methyl)isophthalamide (PhAm) unit into the benchmark polymer donor PM6 for organic photovoltaics.^[^ ^51^ ^]^ The incorporation of PhAm into the PM6 backbone gradually increased the relative intensity of the (0‐0) peak (*I*_0‐0_) to the (0‐1) peak (*I*_0‐1_) in the solution UV–vis profiles and the GIWAXS results of the pristine film indicated **P39** had tighter packing and larger crystals than the reference PM6, which is attributed to improved intermolecular interaction between polymer chains due to H‐bonding from the amide groups in the PhAm unit. Interestingly, the maximum PCEs (PCE_max_s) of the binary OPVs increased from 15.47% (PM6) to 17.45% (**P39** with 10%PhAm) in rigid devices when blending with the nonfullerene acceptor (NFA) Y7, which is caused by the improved charge transport and crystallinity resulting from the intermolecular amide H‐bonding.^[^ ^51^ ^]^

Intrinsically stretchable organic solar cells (IS‐OSCs) with all‐stretchable layers were constructed to compare the photovoltaic and mechanical properties of the blends based on **P39**.^[^ ^51^ ^]^ The initial PCE of the **P39** based IS‐OSC (PCE of 12.73%) was higher than the PM6 based IS‐OSC (PCE of 11.05%) and the strain at PCE_80%_ of the **P39** based IS‐OSC was 32%, whereas it was only 15% for the PM6 based device.

The mechanical properties of the blend films were investigated using pseudo‐free‐standing tensile tests and the PM6:Y7 blend exhibited highly brittle mechanical properties with a COS of only 1.8%, whereas the **P39**: Y7 had a COS of 13.8%. Even at 2% strain, the PM6:Y7 blend showed a sharp crack, while the **P39**:Y7 blend showed plastic deformation with no crack even at 10% strain.^[^ ^51^ ^]^ The **P39** based blend achieved a high PCE while also improving stretchability and this successfully addressed the common trade‐off relationship between these two parameters.

### 2.3 Urea Functional Groups

#### 2.3.1 Overview of the Urea Group and Representative CPs

**Figure 9:**
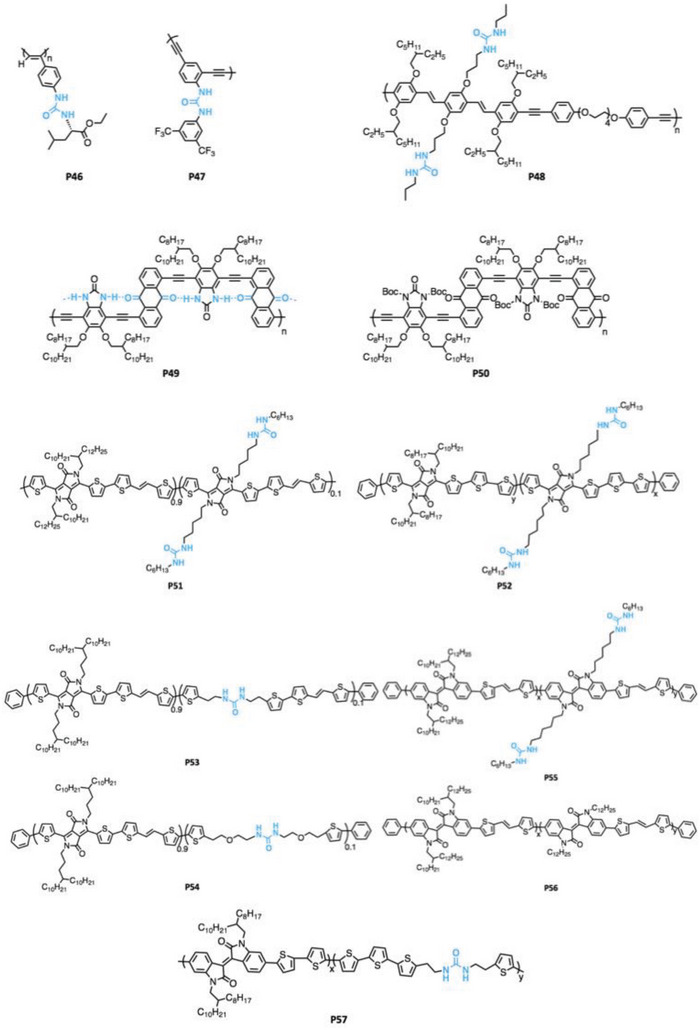
Representative benzene‐based urea functionalized CPs (P46 to P50).

The urea group is well known for strong H‐bonding and directionality because the two N*─*H protons can interact with the oxygen of another carbonyl group. Both lone pairs on the oxygen atom in the carbonyl group can participate in the H bonding to form urea–urea dimers (Figure 1). The urea–urea dimer possesses two relatively stable rotamers with dihedral angles of 0° (coplanar) or 90° (perpendicular).^[^ ^87^ ^]^ Urea–urea dimerization can change interchain interactions, allowing tunability of semiconducting polymer properties and it can also cause polymers to form more organized domains as well as improved polymer domain interconnection.^[^ ^88^ ^]^ The urea–urea dimer is also a sufficient recognition component for a guest molecule to perform the size‐selective molecular recognition.^[^ ^89^, ^90^, ^91^ ^]^ Additionally, the urea functional group can be conveniently installed with a small number of synthetic steps. Similar to amide and carbamate groups, urea groups are most often introduced into the side chain of the CPs and representative urea functionalized CPs are shown in **Figure** **9**.

#### 2.3.2 Synthetic Approaches for Introducing Urea Groups into CPs

Similar to the amide group, the design and synthesis of urea functionalized monomers preceding direct polymerization is the most common strategy. The most typical method to synthesize the urea group is to perform an addition reaction between an amine (R‐NH_2_) and isocynate (R‐NCO), while triphosgene can also be used as the linking moiety to react with an amine (R‐NH_2_) to form the urea group. **P46** was synthesized by the conventional rhodium‐catalyzed alkyne polymerization using the catalyst [Rh(BPh4)(nbd)] with the monomer bearing the urea group.^[^ ^92^ ^]^ **P47** was synthesized via a copper(I)‐mediated oxidative coupling polymerization of the corresponding urea functionalized monomer with a molecular weight of 14 kg mol^−1^.^[^ ^93^ ^]^

**P48**, **P49**, and **P50** were synthesized by Sonogashira polymerization.^[^ ^95^, ^96^ ^]^ **P51‐P57** were polymerized with the Stille polymerization and nearly all the urea groups were incorporated in the aryl‐halogen monomer.^[^ ^88^, ^94^, ^97^, ^98^, ^99^ ^]^ Notably, the *M*_n_ decreased for the Stille‐derived polymers as the ratio of urea monomer increased. For instance, Rondeau‐Gagné et al. showed that the *M*_n_ of **P55** decreased from 13 to 8–9 kg mol^−1^ as the ratio of the urea monomer increased up to 20% mol. More clearly, the weight average molecular weight (*M*_w_) decreased from 60–75 to 18–35 kg mol^−1^, which indicates that the presence of urea is less favorable for forming long chain polymers. Additionally, Fang et al. showed that conducting Sonogashira polymerization directly in the presence of the urea group led to a relatively low *M*_n_ (about 5kg mol^−1^) for **P49**, which is caused by precipitation during solution‐phase synthesis due to the rigid nature of polymer **P49**. Fang et al. adopted the H‐bond masking technique and synthesized the precursor polymer **P50** with high molecular weight (*M*_n_ = 32kg mol^−1^) then converted into a higher molecular weight batch of **P49** in the solid state with thermal cleavage of the Boc protecting group.^[^ ^95^ ^]^

#### 2.3.3 Materials Properties of Urea Functionalized CPs

The application of urea functionalized CPs can be classified across three categories: i) molecular binding of anions ii) solid‐state morphology tuning of CPs, and iii) mechanical property tuning. Anions not only play an important role in biological, industrial, and environmental processes, but are also essential in many areas of chemical research, such as functional materials, transmembrane transport, and catalysis.^[^ ^89^ ^]^ Therefore, selective binding and sensing of anions by synthetic materials has become an important field of supramolecular chemistry. The application of molecular recognition of anions is mainly through the coordination/binding of urea groups with anionic guests and it has been demonstrated that urea can chelate an anion via two directed H‐bonds.^[^ ^90^ ^]^ Use of a CP backbone as a scaffold for the urea anion receptor not only acts as a signaling component that allows for a colorimetric response but can also increase the anion‐binding affinity through the cooperative recognition of multiple spatially arranged urea groups.^[^ ^91^ ^]^ For example, a THF solution of **P46** was pale yellow with an absorption around 400 nm but when tetra‐*n*‐butylammonium acetate (CH_3_CO_2_^−^) was added, the color of the polymer solution immediately became red, demonstrating the polymer's colorimetric response capabilities. The observed color shift was mainly attributed to an increase in the length of the main chain conjugation and the conformational change in the polymer main chain caused by CH_3_CO_2_^−^ binding to the urea receptors. Other anions, such as F^−^, Cl^−^ Br^−^, I^−^, HSO_4_^−^, NO_3_^−^ and N_3_^−^, produced different changes in the absorption.

**Figure 10:**
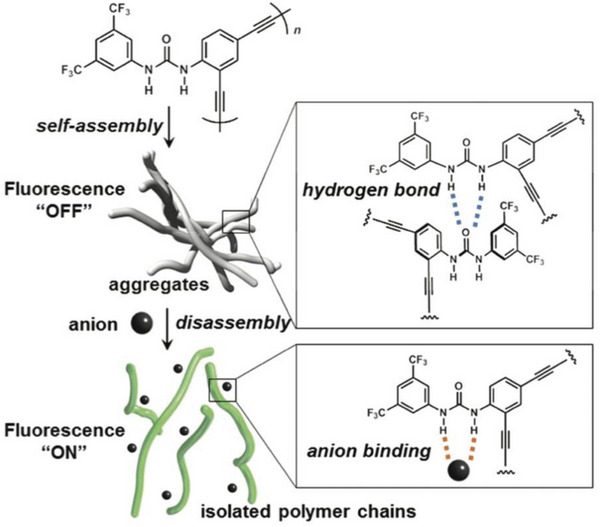
Illustration of fluorescence turn‐on sensing of anions based on the disassembly of **P47**. Reproduced with permission.^[^ ^93^ ^]^ Copyright 2012, American Chemical Society.

**P47** with an alkyne based conjugated backbone exhibited similar anion detection behavior. Here, anion‐recognition prompted disassembly of **P47** aggregates was revealed to be the mechanism of the observed fluorescence turn‐on, as shown in **Figure** **10**.^[^ ^93^ ^]^ To demonstrate this turn‐on fluorescence sensor, Kakuchi et al. measured the fluorescence of **P47** in the presence of various anions and most of the polymer solutions showed intense fluorescence emission when anions were added.^[^ ^93^ ^]^

Urea groups have also been inserted into CPs in both the backbone and in the side chain to tune morphology and crystallinity. Bout et al. synthesized **P48** and investigated the folding of the CPs at the single molecule level.^[^ ^96^ ^]^ Based on single molecule excitation polarization spectroscopy, it was found that urea‐containing side‐chains have higher folding order and it was hypothesized that the redshift of 0.06 eV in the 0‐0 absorption peak for the urea‐containing polymer is due to the backbone planarization caused by a highly organized urea‐containing polymer structure. Fang et al. tuned the solubility between **P49** and **P50** by chemically blocking and rebuilding the preorganized intramolecular hydrogen bonds, which resulted in a stiff ladder‐type conformation for **P49** from the precursor **P50** in thin films via in situ thermal treatment.^[^ ^95^ ^]^ Zhang et al. reported the synthesis of **P52** and found that adding urea groups in the alkyl side chains improved OFET hole mobility after thermal annealing.^[^ ^88^ ^]^ It worth noting that the mobility increased by incorporating more urea group and **P52** with about 10 mol% urea, gave the highest mobility of 13.1 cm^2^ V^−1^ s^−1^. Zhang et al. ascribed this improvement to the increased lamellar packing order of the alkyl chains where each has the urea group and modest inter‐chain stacking. Introducing hydrogen bonding into the side chain of DPP polymers to construct high performance OFETs is a widely explored approach. The amide based DPP polymer (**P33**) achieved a maximum hole mobility of 2.46 cm^2^ V^−1^ s^−1^, while the urea based DPP polymer (**P52**) enabled an impressive hole mobility of 13.1 cm^2^ V^−1^ s^−1^. This is perhaps due to the stronger and more directional hydrogen bonding in the urea group when compared to the amide group, leading to a higher crystallinity and more ordered structure in the thin film state, thus strongly enhancing charge transport. However, the higher crystallinity induced by the introduction of urea group might be detrimental to the mechanical properties, while the amide‐based polymers might avoid this potential trade off.

More interestingly, introducing urea groups into the alkyl side chains also has a positive impact on the photovoltaic performance of the blend with **P52** donor and PC_71_BM acceptor. Zhang et al. found that the urea groups may help **P52** assemble into nanofibers and the PC_71_BM acceptor aggregate in a more ordered fashion, as proven by the microphase separation observed in AFM images. Therefore, the **P52**: PC_71_BM blend exhibited PCE between 6 and 7%, while most of the reference and control polymers gave PCE close to or lower than 5%.^[^ ^88^ ^]^ Similarly, Rondeau‐Gagné et al. synthesized **P55** by inserting the urea group into the side chain of the isoindigo‐based polymers (**P56** with linear hydrocarbon side chain as a control) and observed the trend that the OFETs made from polymer with 20% urea moieties had higher average hole mobility (0.032 cm^2^ V^−1^ s^−1^) than **P56** with 20% dodecyl side chains (0.0073 cm^2^ V^−1^ s^−1^).^[^ ^98^ ^]^ Additionally, Deshmukh et al. also introduced the urea group as a conjugation break spacer (CBS) into the backbone of the isoindigo‐based polymers (**P57**).^[^ ^99^ ^]^

Since the urea group enables H‐bonding, the impact of the urea group on mechanical properties of CPs is also very interesting. Bao et al. synthesized **P53** and **P54** to investigate the impact of urea on the mechanical properties of DPP‐based CPs and found that the CPs with urea groups generated greater polymer chain aggregation and crystallinity in thin films, which lead to a higher modulus and crack on‐set strain.^[^ ^94^ ^]^ Furthermore, the rDoC (relative degree of crystallinity) of the stretched thin film with the greatest crack on‐set strain experienced nearly no decrease in the ratio, indicating the predominant energy dissipation process is the breaking of dynamic H‐bonds. On the other hand, other less stretchy polymer films based on the amide analogues, released the strain energy by breaking the crystalline domain, as demonstrated by a significant decrease in rDoC.^[^ ^94^ ^]^ **P54** exhibited slightly higher modulus than **P53** which might be due to more oxygen atoms that potentially can participate in H‐bond formation.

Importantly, Bao et al. also summarized four strain energy dissipation mechanisms in H‐bonding functionalized CPs: 1) breakage of H‐bonding sites, 2) reorientation and alignment of crystalline domains, 3) chain extension and alignment in amorphous regions, 4) breaking of crystalline domains.^[^ ^94^ ^]^ In addition, both **P53** and **P54** have higher mobility than the control polymer without H‐bonding because of their substantially higher crystallinity and distributed H‐bonding domain caused by the urea group. **P53** and **P54** also demonstrated the ability to maintain charge transport properties in fully stretched transistors. Interestingly, Gu et al. investigated the urea side chain incorporated DPP polymer **P51** and discovered a significant difference in ductility where urea functionalization leads in a 50% loss in strain at failure.^[^ ^97^ ^]^ **P51** exhibited an impressive crack onset (COS) of ≈50% with the film‐on‐elastomer (FOE) measurement while a low COS with the film‐on‐water (FOW) measurement.^[^ ^97^ ^]^ Depending on the measurement conditions, **P51** displayed both low and high ductility, which suggests that two competing mechanisms—potentially high crystallinity, which reduces ductility, and energy dissipation via hydrogen bonding, which increases ductility—determine its mechanical performance.^[^ ^97^ ^]^ **P51** was found to experience a fast‐initial swelling in the aqueous environment and the strong hydrogen bond interaction of urea moieties with water is assumed to be the reason of the initial fast swelling.

Due to the symmetrical and triatomic intermolecular geometry between urea moieties,^[^ ^94^ ^]^ they can produce strong H‐bonding interaction energy, which can cause directed crystallization and also possibly cause a lack of stress tolerance if the plasticization of the urea group with water molecules happens. It worth highlighting the stark mechanical difference between two urea incorporated DPP polymers **P51** (side chain) and **P53** (backbone).^[^ ^94^, ^97^ ^]^ Furthermore, it should be considered that in order to incorporate the urea group into the backbone of DPP CPs, **P53** consequently has certain content of conjugation break spacer which can be an important variable that needs to be considered since breaking the conjugation generally will lead to lower elastic modulus and improved ductility.^[^ ^100^, ^101^, ^102^, ^103^, ^104^, ^105^, ^106^, ^107^, ^108^ ^]^ Overall, this stark mechanical difference also offers a good lesson that the future of intrinsically stretchable CP design needs to be carefully evaluated since side chain engineering and backbone engineering using the same urea group can bring completely opposite results.

### 2.4 Thymine Functional Groups

#### 2.4.1 Overview of the Thymine Group and Representative CPs

**Figure 11:**
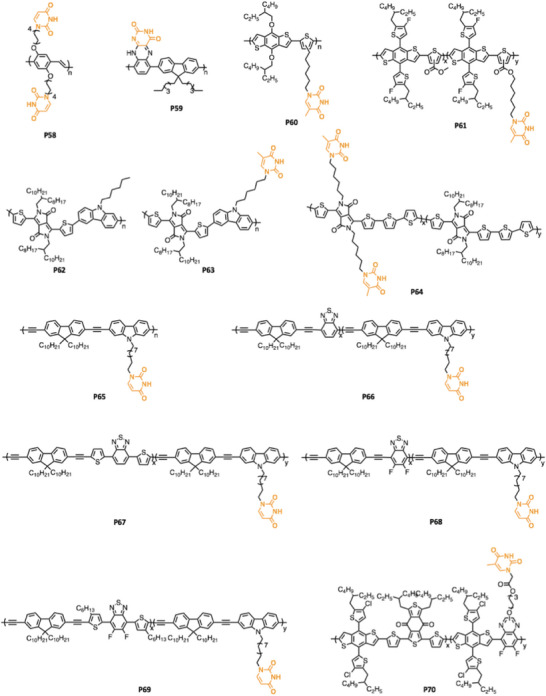
Representative thymine functionalized CPs (P58–P70).

**Figure 12:**
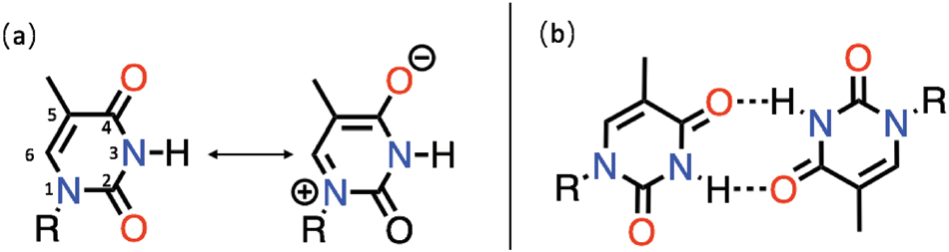
a) Illustration of thymine resonance structures and b) the thymine–thymine dimer.

Thymine is a well‐known nucleobase in the nucleic acid DNA. Inspired by biological macromolecules, the introduction of thymine into synthetic polymers is an interesting molecular design strategy (**Figure** **11**). Importantly, the N‐3 proton of thymine is more acidic than the N*─*H proton of an amide group due to the two adjacent carbonyl groups rendering this an imide type functionality. Additionally, the basicity of C═O bond which participates in the dimerization at the C‐4 of thymine, is stronger than the typical carbonyl group since the oxygen bonded to C‐4 is conjugated with N‐1 via C‐5, C‐6 double bond according to its resonance structure, as shown in **Figure** **12**. These effect induce thymine to have a significantly stronger tendency to self‐dimerize (dimerization constant about 15 m ^−1^) than other common H‐bonding functional groups that have been widely adopted into CPs (i.e., amide group with dimerization constant about 5 m ^−1^).^[^ ^109^ ^]^ Nearly all thymine functionalized CPs have been synthesized by direct polymerization with the thymine functionalized monomer and in all cases, the thymine unit was introduced into the side chain in order to maintain the conjugation of the backbone and maximize the H‐bonding interaction.

#### 2.4.2 Synthetic Approaches for Introducing Thymine Groups into CPs

**Figure 13:**
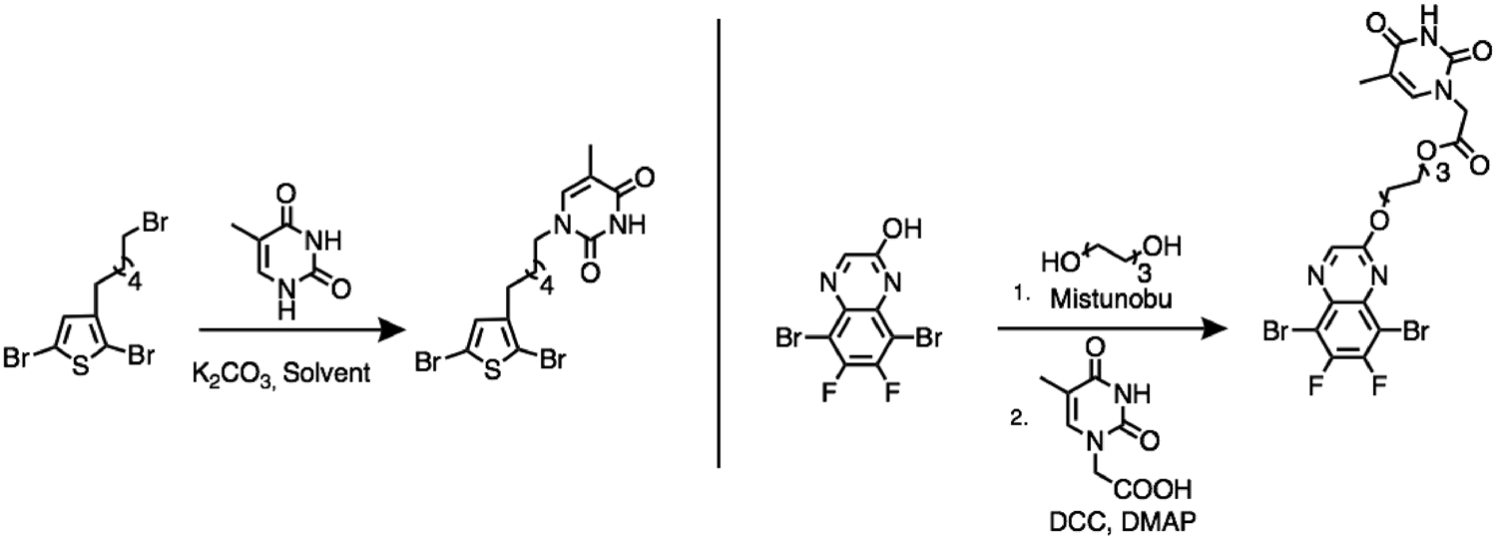
Two representative thymine functionalized monomer syntheses.^[^ ^110^, ^117^ ^]^

As direct polymerization of thymine functionalized monomers is the dominant approach, synthetic strategies can be subdivided into two steps: i) monomer design and synthesis and ii) polymerization. The thymine functionalized monomers for P58, P60, and P63‐69 were synthesized using traditional S_N_2 reactions which involve the reaction between an alkyl‐halide and thymine (5‐Methylpyrimidine‐2,4(1H,3H)‐dione).^[^ ^55^, ^110^, ^111^, ^112^, ^113^ ^]^ Interestingly, Yamaguchi et al. synthesized the thymine analogous monomer (alloxazine‐6,9‐diyl unit) of P59 by a condensation between the alloxane and 1,4‐dibromo‐2,3‐diaminobenzene.^[^ ^114^ ^]^ Son et al. synthesized 1‐(6‐hydroxyhexyl)−5‐methylpyrimidine‐2,4(1H,3H)‐dione as the thymine source and performed the Steglich Esterification with 2,5‐dibromothiophene‐3‐carboxylic acid to synthesize the thymine functionalized monomer for P61.^[^ ^115^ ^]^ Thompson et al. designed the thymine side chain terminated 6,7‐difluoro‐quinoxaline (Q‐Thy) monomer based on the benchmark acceptor unit 5,8‐dibromo‐6,7‐difluoroquinoxalin‐2‐ol^[^ ^116^ ^]^ starting with the most commonly used thymine source in the supramolecular field (thymine‐1‐acetic acid) and synthesized P70.^[^ ^117^ ^]^ Two representative synthetic routes for the synthesis of thymine functionalized monomers are shown in **Figure** **13**. It has been demonstrated that using thymine‐1‐acetic acid as the thymine source rather than direct alkylation can avoid side reactions and purification issues caused by tautomers, such as N‐3 alkylation and O‐alkylation products, potentially significantly widening the scope of substrates.^[^ ^118^, ^119^ ^]^

**P61**, **P64**, and **P70** were synthesized by the Stille polymerization and **P65**‐**P69** were synthesized by Sonogashira polymerization. In general, the *M*_n_ of terpolymers starts decreasing as the ratio of thymine functional monomer is increased. For instance, the *M*_n_ of **P64** decreased from 95 to 30 kg mol^−1^ with 5 mol% loading of thymine monomer.^[^ ^112^ ^]^ The *M*_n_ of **P70** decreased to the half of the original *M*_n_ without thymine when the incorporated ratio of thymine monomer was 20%.^[^ ^117^ ^]^ The *M*_n_ of **P61** significantly decreased from 52.6 to 11.9 kg mol^−1^ as the ratio of thymine monomer increased from 0% to 20%.^[^ ^115^ ^]^ It is suggested that the decreased *M*_n_ of the terpolymers could be mainly attributed to palladium‐thymine interference during polymerization.^[^ ^120^ ^]^ It is also worth highlighting that with **P58**, **P59**, and **P60** every repeat unit has a thymine group and reasonable molecular weights were still achieved.^[^ ^55^, ^110^, ^114^ ^]^ Interestingly, in these case, all were polymerized in polar solvents. For instance, polymerization of **P58** was in THF, **P59** was on THF/NMP and **P60** was in DMF.

#### 2.4.3 Materials Properties of Thymine Functionalized CPs

Similar to the urea group, the application of thymine functionalized CPs can be classified into three categories: i) molecular recognition, ii) morphology tuning, and iii) mechanical impact.

Since one of the signature advantages of thymine is molecular recognition with a complementary H‐bonding unit such as adenine, utilizing thymine functionalized CPs to identify other moieties with corresponding complementary H‐bonding is very common. For instance, Wang et al. synthesized a novel CP nanogel carrier based on **P58** and successfully developed triple hydrogen bonded drug conjugation, which allowed long‐term drug release with improved drug‐loading efficiency, stability, and biocompatibility.^[^ ^55^ ^]^ Qin et al. also investigated the impact of molecular recognition between thymine based P3HT analogous polymers and diaminopyridine functionalized fullerene.^[^ ^121^, ^122^, ^123^ ^]^

Yamaguchi et al. found that **P59** has photoluminescence in solution at 581 nm and that photoluminescence decreased with the addition of nucleosides including adenosine (A) and guanosine (G) and with metal ions such as Cu(I), Cu(II), and Zn(II).^[^ ^114^ ^]^ It was proposed that the drop of PL intensity caused by the addition of nucleosides and metal salts is very likely due to electron transfer from the originally excited polymer to the complexes formed by hydrogen bonding between the polymer alloxazine unit and the nucleoside. Zhang et al. found Pd(II) and Hg(II) ions can be independently integrated into **P64** polymer thin films via air–water interface coordination and that FETs based on these thin films responded sensitively and selectively to CO and H_2_S, respectively.^[^ ^112^ ^]^ It worth highlighting the CO with a low concentration of 10 ppb can be detected by **P64**‐Pd(II)‐based FETs which only incorporate 5 mol% thymine monomer, whereas H_2_S with a concentration of 1 ppb can be detected by FETs using **P64**‐Hg(II) thin films.^[^ ^112^ ^]^

Due to the strong tendency of thymine to dimerize, introducing thymine into the side chain of CPs generally has a significant impact on the solid‐state morphology. Zhang et al. compared **P62** and **P63** and found that H‐bonding from thymine facilitated assembly into highly ordered structures, which improved intermolecular charge transfer and enabled a nearly five‐times higher hole mobility as compared to the polymer without the thymine group.^[^ ^111^ ^]^ More interestingly, Zhang et al. observed a broad N*─*H peak between 3100 and 3680 cm^−1^ in the IR and found the magnitude of the broad peak dramatically increased after 5 min of thermal annealing at 140 °C, which indicates the as‐cast **P63** film has free thymine groups which become H‐bonded during thermal annealing. Walter et al. found that the inclusion of thymine significantly increased hole mobility as **P60** has a hole mobility of µ_h_ of 7.2 × 10^−6^ cm^2^ V^−1^ s^−1^ which is significantly higher than the alkyl side chain control polymer with the µ_h_ of 3.9 × 10^−8^ cm^2^ V^−1^ s^−1^. The observed improvement in mobility was ascribed to close and well‐organized packing.^[^ ^110^ ^]^

Jia et al. revealed that the incorporation of physical crosslinking based on thymine H‐bonding can assist self‐assembly, suppressing severe aggregation of chromophores in thin films and provides improved electroluminescent performance in PLEDs.^[^ ^113^ ^]^ As such, the photoluminescence and electroluminescence of **P65**‐**P69** were greatly improved over non‐thymine control polymers. Zhang et al. also observed that **P64** has higher mobility (9.1 × 10^−6^ cm^2^ V^−1^ s^−1^) than the analogous CP without thymine and ascribed this enhancement to the improved crystallinity.^[^ ^112^ ^]^ Similar to the trend between amide and urea, a relatively stronger hydrogen bonding interaction in a thymine‐based DPP polymer (**P64**) also exhibited a higher maximum hole mobility of 9.1 cm^2^ V^−1^ s^−1^ compared to the amide based DPP polymer (**P33**). The GIWAXS results show that the inclusion of thymine groups in the side chains improves the lamellar packing. For instance, scattering signals up to the fourth order were seen in the out‐of‐plane direction for **P64** thin films due to lamellar stacking of side chains while, in comparison, the corresponding (100) and (200) signals for the nonthymine control polymer thin films were found to be weak and broad. Furthermore, the lamellar stacking signals for **P64** thin films are sharper than for the pure alkyl side chain polymer thin films and with a smaller full width at half‐maxima. In fact, the improved crystallinity is most likely due to the development of H‐bonding between the thymine groups, which also causes the polymer chains to pack more tightly.

Since thymine enables strong dimerizable H‐bonding, the impact of thymine on the mechanical properties of CPs is also gaining significant attention. Son et al. found the COS of neat polymer **P61** significantly increased as the ratio of the thymine incorporated monomer increased based on the film‐on‐elastomer method.^[^ ^115^ ^]^ The crystal coherence length *L*_C(100)_ and *L*_C(200)_ values for **P61** blend films with the nonfullerene acceptor IT4F^[^ ^124^ ^]^ are clearly higher than the *L*_C(100)_ and *L*_C(200)_ values of the blend film using the nonthymine control copolymers, which is likely the result of H‐bonding between the thymine units. However, the PCE in OPV of the **P61** blend film decreased from 13.4% to less than 12% as the ratio of the thymine incorporated monomer increased in the polymer. Importantly, as thymine content was increased the molecular weight significantly decreased from about 50 to about 10 kg mol^−1^, which can have significant impact on the PCE since a significantly lower *M*_n_ can cause a different blend morphology, molecular packing and domain sizes.^[^ ^125^, ^126^ ^]^

Also focusing on thymine‐functionalized CPs in OPV, Thompson et al. observed a similar trend with a blend of **P70** and small molecule NFA L8‐BO^[^ ^127^ ^]^ showing significantly larger *L*_C (200)_ (13.0 nm), *L*_C(010)_ (2.9 nm), and smaller *d*_010_ (3.72 Å) than the blend with PM7 control polymer without thymine ^[^ ^128^ ^]^ which showed *L*_C (200)_ of 8.4 nm, *L*_C(010)_ of 2.3 nm, and a π–π stacking distance *d*_010_ of 3.79 Å.^[^ ^117^ ^]^ More interestingly, the **P70** blend exhibit a higher PCE than the PM7 blend and this is attributed to improved crystallinity in **P70** enable by thymine induced H‐bonding and better mixing between **P70** and the acceptor in the blend, which leads to enhanced charge generation, higher hole mobility and PCE.^[^ ^129^, ^130^ ^]^ Film‐on‐water measurements were conducted to evaluate the impact of thymine functionalization on the mechanical properties of the blend.^[^ ^117^ ^]^ The **P70** blend exhibited a COS of 13.7% and a toughness of 4.5 MJ m^3^, which are 5 and 9 times greater than the COS and toughness of the PM7 blend, respectively. Additionally, film‐on‐elastomer with TPU/PEDOT:PSS/active layer architecture has been measured and the **P70** based blend exhibited significantly higher mechanical durability.

**Figure 14:**
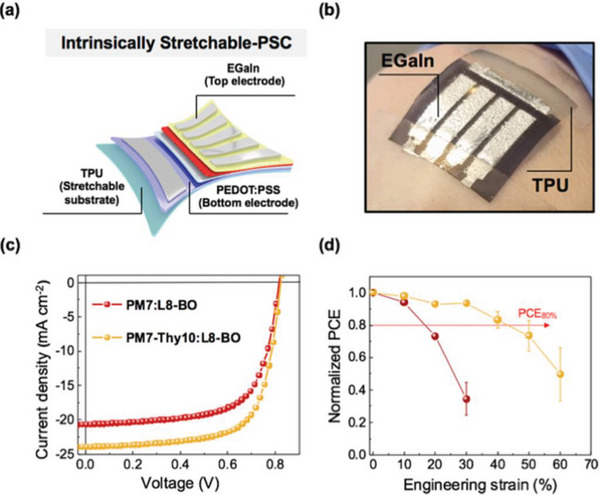
a) Device structure and b) image of the intrinsically stretchable PSC (IS‐PSC). c) *J*–*V* curves of the IS‐PSCs with PM7 and PM7‐Thy (**P70**)‐based blends. d) Normalized PCE of IS‐PSCs during stretching. Reproduced with permission.^[^ ^117^ ^]^ Copyright 2023, American Chemical Society.

In order to highlight the improved mechanical properties, the **P70** blend was introduced to an intrinsically stretchable polymer solar cell (IS‐PSC) with a TPU/PEDOT:PSS/active layer/Interfacial layer/EGaIn device architecture. Prior to stretching, the **P70**‐based IS‐PSC outperformed the PM7‐based IS‐PSC with PCE of 13.7%, owing to increased *J*_sc_ and FF. The PCE of the PM7‐based IS‐PSC decreased sharply at 10% strain, and the strain at PCE_80%_ was 16.5%, while the PM7‐Thy10‐based IS‐PSC, demonstrated much improved stretchability with a strain at PCE_80%_ of 43.1%, as shown in **Figure** **14**.

Interestingly, both the amide based polymer (**P39**) and thymine based polymer (**P70**) were examined in OPV devices. A Similar trend in enhanced crystallinity in the NFA blends were observed in both cases. **P70** has the stronger H‐bonding thymine functional group but it worth noting that although **P39** is an amide based polymer, it has two equivalents of H‐bonding group relative to **P70**. As a result, both polymers exhibited improved intermolecular interaction that led to a tighter packing and larger crystals in the blend and benefited the PCE. More encouragingly, both polymers showed significantly improved COS in the blend that might indicate that they are subject to the strain energy dissipation mechanism for breakage of H‐bonding sites.

In a related approach, Son et al. introduced melamine into a **P61** blend with NFA IT4F to investigate the self‐healing property via molecular cross linking between thymine and melamine.^[^ ^115^ ^]^ One melamine molecule can bind with three thymine groups and this enables self‐healing since self‐healing occurs via polymer matrix reorganization, which is accompanied by the regeneration of dynamic bonds, such as H‐bonds and the tangling of polymer chains at the damaged interfaces.^[^ ^131^ ^]^ Specifically, nanocracks in **P61** based BHJ films disappeared after adding the melamine with the assistance of thermal treatment. However, the PCE of the corresponding self‐healable blends decreased from 13% to less than 11%, which also indicates the difficulty in addressing the trade‐off between outstanding photovoltaic performance and excellent self‐healing properties. Therefore, the performance of self‐healable OPV needs to be improved further with the goal of obtaining a high photovoltaic performance (i.e., PCE > 17%) as well as an excellent self‐healing property (excellent maximum recoverable strain and PCE) simultaneously.

## 3 Other H‐Bonding Functional Groups Used in CPs

**Figure 15:**
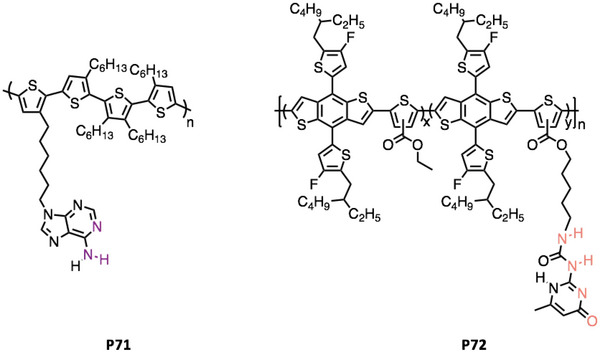
Representative Upy and adenine based CPs.

Due to very limited examples of the corresponding H‐bonding functionalized CPs, other H‐bonding CPs based on groups such as adenine and ureidopyrimidone are briefly reviewed in this section. Adenine, also a well‐known nucleobase in the nucleic acid DNA, has been introduced into CPs. For instance, Walter et al. introduced adenine into benzo[1,2‐b:4,5‐b’]‐dithio‐phene (BDT) based CPs and this led to a significant redshift in absorption due to the strong molecular assembly.^[^ ^110^ ^]^ Kilbey et al. synthesized an adenine‐functionalized thiophene‐based alternating copolymer via Direct Arylation Polymerization.^[^ ^132^ ^]^ It worth highlighting that the primary amine in adenine was protected by the Boc group since adenine can strongly bind with the palladium metal center. Kilbey et al. found the interchain H‐bonding via adenine can significantly improve the packing of the copolymer, which resulted in a 70 °C increase in glass transition temperature compared to the unfunctionalized control polymer. Additionally, the nucleobase's ability to bind heavy metal ions resulted in a substantial fluorescence suppression (>90%) upon addition of Cu^2+^ ions, which represented a high Stern–Volmer constant. Subsequently, Kilbey et al. conducted a facile one‐pot synthetic method including Direct Arylation Polymerization followed by Boc deprotection to create an adenine‐containing poly(alkylthiophene) **P71** (**Figure** **15**) by careful temperature control, which eliminated unnecessary purification and isolation steps and allowed the overall synthesis to become more efficient and feasible with the production of higher molecular weight polymers.^[^ ^133^ ^]^ Jiang et al. introduced adenine into the side chain of a fluorene‐based polymer and found that the adenine unit could improve the interaction between the surface of the polymer photocatalyst and water molecules by forming hydrogen bonds, which significantly increased the hydrophilicity and distribution of the resulting polymer photocatalyst in the photocatalytic reaction solution.^[^ ^134^ ^]^ As a result, the adenine‐functionalized fluorene polymer exhibited a high photocatalytic activity under UV–vis irradiation with a hydrogen evolution rate (HER) of 25.21 mmol g^−1^ h^−1^, which is significantly higher than the control polymer without the adenine group (6.53 mmol g^−1^ h^−1^).^[^ ^134^ ^]^ More impressively, the fluorene based polymer with adenine demonstrated an outstanding HER of 21.93 mmol g^−1^ h^−1^ under visible light (*λ* > 420 nm) without the inclusion of a Pt cocatalyst.

Since Meijer et al. first reported the ureidopyrimidinone (Upy) unit in 1997,^[^ ^135^ ^]^ the UPy unit and its derivatives have been introduced to many fields based on the advantages of ease of synthesis, available starting materials and high association constant.^[^ ^136^, ^137^, ^138^, ^139^, ^140^ ^]^ For example, Son et al. introduced the Upy unit into the side chain of a BDT‐based polymer **P72** (Figure 15) and found that H‐bonding between polymer side chains can efficiently maintain the ordered polymer packing patterns under applied strain.^[^ ^141^ ^]^ As such, the TFT device with Upy maintained a relatively high mobility of 5.8 × 10^−3^ cm^2^ V^−1^ s^−1^ in the direction perpendicular to the strain direction due to the increased crystallinity of the polymer, whereas the device based on the control polymer showed a dramatic decrease in hole mobility under strain from 1.0 × 10^−2^ to 5.6 × 10^−3^ cm^2^ V^−1^ s^−1^ due to morphological changes and cracking of the film.^[^ ^141^ ^]^ Verduzco et al. synthesized Upy‐terminated CPs by reacting hydroxyl or primary amine terminated parent CPs with reactive isocyanate Upy group and found that the quadruple hydrogen bonding interactions can be exploited to prevent micrometer‐scale phase separation within the blend consisting a CP and an insulating polymer under thermal annealing.^[^ ^142^ ^]^ Specifically, different from the unmodified polymer blends, the blends of UPy‐terminated CPs do not exhibit micrometer‐scale phase separation even after extended thermal annealing (i.e., 160 °C with 16 h). Additionally, photoluminescence experiments indicate the UPy modification can promote the PL quenching in the donor and acceptor polymer solutions, owing to hydrogen‐bonding associations that lower the average distance for energy and electron transfer. Verduzco et al. further investigated the photovoltaic performance of the Upy‐terminated polymer‐polymer blend and found a slight increase in power conversion efficiency.^[^ ^143^ ^]^ Impedance analysis of polymer‐blend OPVs identified an increase in both charge transport and charge recombination resistance in the annealed OPVs, which indicates the UPy modification strategy can be an effective approach to reduce macrophase separation, optimize the interfacial electrical characteristics in polymer/polymer blends and generate high‐performance photovoltaic devices. Qiu et al. inserted the ring‐like quadruple H‐bonding UPy unit into the backbone of a DPP‐based polymer resulting in the breakdown of the backbone's conjugation with simultaneous strong H‐bond inclusion, which improved the mechanical properties.^[^ ^144^ ^]^

Fluorination is a widely used design strategy in the CP field.^[^ ^145^ ^]^ As an electronegative atom, fluorine can act as the hydrogen bond acceptor and there exist examples where fluorine atoms on CPs participate in the hydrogen bonding.^[^ ^48^ ^]^ However, the fluorine atom on the CPs will mostly likely affect the conformation of CPs via the secondary interaction rather than actual hydrogen bonding.^[^ ^146^, ^147^, ^148^, ^149^, ^150^, ^151^, ^152^ ^]^ Therefore, this review is not focus on fluorine‐based CPs.

Carboxylic acids are one of the hydrogen bonding functional groups that have been introduced into CPs. Katz et al. found that carboxylic acid‐functionalized amphiphilic polythiophenes exhibited a higher stability and better pH sensitivity than P3HT in aqueous solution.^[^ ^153^ ^]^ You et al. reported a random polythiophene with 50% mol% thermocleavable tertiary ester side chain.^[^ ^154^ ^]^ After thermal annealing treatment, which led to the cleavage of the tertiary ester group, the carboxylic acid based polythiophene demonstrated significantly improved π–π stacking, which led to greater charge mobility. Further, these carboxylic acid functional polythiophenes in the solid state have considerably enhanced stretchability and the sensors based on these carboxylic acid polythiophenes not only detect humidity and ethanol but also light and heat energy owing to the hydrogen bonding resulting from the carboxylic acid group. Additionally, carboxylic acid groups are critical in designing CPs for dye‐sensitized solar cells because the CPs must adhere to the nanostructured TiO_2_ surface via the interaction between the polar carboxylic acid units with the metal oxide surface.^[^ ^155^ ^]^ Overall though, carboxylic acid based CPs have limited examples and applications and therefore this review is not focus on carboxylic acid based hydrogen bonding in CPs.

## 4 Conclusion and Outlook

Different types of H‐bonding functional groups used in the CP field and representative CPs have been presented. The synthetic methods for introducing the corresponding H‐bond functional groups into CPs have also been highlighted. Generally, introducing H‐bonding into CPs increases the hydrophilicity. Further, the creation of strong noncovalent H‐bonds within polymer thin films results in not only in more ordered and crystalline structures due to the molecular assembly, but also the powerful aggregation and enhanced packing, which can benefit charge transport within and between polymer chains. Therefore, H‐bonded CPs with excellent charge‐transport properties have been achieved. Additionally, H‐bonding groups distributed in the polymer films can act as physical crosslinking sites due to the reversible nature of the noncovalent bonds, which can significantly impact the mechanical properties of polymer films. It has been demonstrated that it is possible to address the trade‐off between mechanical robustness and electronic or photovoltaic performance by utilizing the H‐bonding strategy.

Looking forward, several aspects of H‐bonding CPs need to be expanded upon to further realize the full potential impact of this approach. Specifically: i) A greater variety of different types of H‐bonding groups should be considered since the majority of the H‐bonding strategies in CPs are limited to the secondary amides, carbamates, and urea groups. Although H‐bonding groups have been demonstrated as a successful design strategy for certain applications, other H bonding groups such as triple and quadruple H‐bonding groups could offer greater control over properties. For instance, the UPy unit has a significantly higher association constants than most of the hydrogen bonding groups reported for use in CPs. It will be interesting to thoroughly understand the impact of the UPy unit on the morphology, aggregation, electrical, and mechanical properties of CPs. Additionally, systematically investigating hydrogen bonding groups with different strengths but on identical polymer backbones will offer better understating of the impact of hydrogen bonding groups on fundamental properties and in the context of varying applications. ii) The scope of the substrate where H‐bonding group are attached needs to be significantly expanded. The CP field has a broad diversity of monomers ranging from strong electron donating units to the strong electron deficient units. However, nearly all the substrates with H‐bond groups are based on very simple moieties such as thiophene, fluorene (or carbozole), and diketopyrrolopyrrole. Only a very few existing examples such as the 6,7‐difluoro‐quinoxaline (Q‐Thy) are based on state‐of‐the‐art monomer units. Some benchmark moieties such as C8‐BTBT, naphthalene diimide, fluorobenzotriazole (FTAZ), NFAs such as Y6 and its derivatives should be considered since the functionalization of benchmark units with hydrogen bonding groups is an underutilized approach. iii) Successfully polymerizing high molecular weight CPs with H‐bonding groups is still challenging regardless of the transition metal cross‐coupling used (Stille, Suzuki, Sonogashira, and DArP). Molecular weight, as one of the intrinsic and decisive factors, plays a key role in determining the properties of CPs. Although solubility is a concern for high molecular weight CPs, most of the H‐bonding functionalized terpolymers are not able to reach this stage because the H‐bonding groups are very likely to interact with the transition metal catalyst and interfere with the catalytic cycle during polymerization. It is still very challenging to develop a general direct polymerization condition than can overcome this drawback. Systematic investigations of polymerization conditions including optimizing the diverse methods (i.e., Suzuki, Stille, DArP, and postpolymerization) and the details such as catalysts, ligands, and solvents are needed. iv) More elucidation and understanding of the role and impact of the H‐bonds in CPs is needed to assist future molecular design. For instance, introducing H‐bonds into a DPP‐based polymer decreased the modulus while an increased modulus was observed in other polymers. Moreover, introducing urea groups into the side chain versus the backbone of DPP polymers caused two completely opposite results. v) More aspects of existing applications should receive greater attention rather than only focusing on the impact on morphology and mechanical properties. For instance, utilizing the coordination between anion and specific hydrogen bond to construct CP‐based chemical sensors. Additionally, introducing hydrogen bonding into CPs generally will improve the hydrophilicity and this might benefit CPs when utilized as the photocatalyst for water splitting. Overall, H‐bonding groups have shown tremendous potential to tailor electronic and physical properties in CPs. However, there is much work to do to further elucidate structure–property relationships and to fully and broadly exploit the potential of this approach.

## Conflict of Interest

The authors declare no conflict of interest.
